# Complex Regional Pain Syndrome Post COVID-19 Vaccine Shot: An Autobiographical Case Report

**DOI:** 10.7759/cureus.20257

**Published:** 2021-12-08

**Authors:** Praveena Raman

**Affiliations:** 1 Oral Medicine and Radiology, Sathyabama Dental College and Hospital, Chennai, IND

**Keywords:** covid-19 vaccine, neuropathic pain syndrome, reflex sympathetic dystrophy syndrome, complex regional pain syndrome, autobiographical case report

## Abstract

Complex regional pain syndrome (CRPS) is a pathological exaggeration caused by trauma from injections and vaccine administration characterized by severe pain (often burning), hyperesthesia, allodynia, edema, vasomotor changes, decreased range of motion, hyperhidrosis, and trophic changes. It occurs at all ages with female predominance, and the incidence increases until late middle age. Hand and foot involvement is well recognized, and this may spread proximally. Treatment usually requires a multimodal approach, including medications and physical and cognitive therapy. Reports of CRPS after vaccination are rare. The incidence of CRPS post coronavirus disease 2019 (COVID-19) vaccination is not yet reported. This case report describes my experience with deltoid hematoma, tennis elbow, and living with CRPS post COVID-19 vaccine shot, including the psychosocial adaptations I made in my day-to-day life.

## Introduction

Diagnosis of complex regional pain syndrome (CRPS) postinjection is uncommon and characterized by hyperalgesia or allodynia, edema, weakness, and vasomotor malfunction according to the inducing cause [1]. The other older terminologies used to describe CRPS include “causalgia” and “reflex sympathetic dystrophy” (RSD) [1]. The etiology is unclear; however, early symptom identification and treatment might minimize complications and total pain as there is no gold standard diagnostic modality specific for CRPS [2]. Among the most difficult neuropathic pain syndromes to treat is complex regional pain syndrome (CRPS), a condition so diffuse and poorly defined that its very existence has recently been called into question [2].

Coronavirus disease 2019 (COVID-19) has caused a significant impact on every human on earth, specifically on all healthcare professionals. Several vaccines were introduced in the market to mitigate this pandemic disease. Initially, the public carried a stigma toward vaccines, and gradually, the purpose and the benefit of vaccines got acquainted among them.

This case report narrates my experience of living with CRPS post COVID-19 vaccine shot, with impact on my lifestyle and quality of life. My goal in writing this autobiographical case report is to provide a resource to my colleagues regarding neuropathic pain post needle pricks and to inculcate compassionate care toward sufferers especially if it is iatrogenic.

## Case presentation

I am an active 33-year-old wife, a dentist of 10 years, a self-practicing oral medicine specialist for two years, and a faculty member at a Local University for 4.7 years before injuring my left hand eight months ago after taking the COVID-19 vaccine shot. I took the first shot on 30th April 2021. On the same night, I developed a high fever with chills, body ache, heaviness on the left hand at the injection site, and severe left-hand pain. As there were many reports on post COVID-19 vaccine fever and tiredness, I did not take it that seriously, thinking that my symptoms are quite normal and I should be feeling better within two days. My fever persisted until 2nd May 2021, and I started developing on/off episodes of increased sweating, along with body pain and left-hand pain with raised temperature and swelling involving the injection site at the deltoid region extending downward to my left elbow, left wrist, and phalanges, causing decreased range of motion. The edema was also associated with itching and some kind of irritation on my entire left hand, and the irritation was severe at the injection site, at the left elbow, on my left palm, and between fingers. The heaviness and pain on the entire left hand were so severe that I was unable to raise my left hand to tie my hair with a band, and I was unable to do my activities of daily living (ADLs), including toileting, wearing my own pants, opening my own water bottle independently (I was opening the bottle lid using my right hand by holding the bottle between my thighs), switching on fans, holding a vessel in kitchen, and even operating a phone/laptop. I lost the total power/strength in my left fingers as I was not having a grip to hold a pen, a spoon, and my house key, as well as switching on the gas knob and doorknob and holding the soap while bathing. I was also not able to turn toward my left side while sleeping due to pain and swelling at the injection site and was struggling with episodes of sleeplessness causing headaches. The episodes of pain were initially mixed with paresthesia, dysesthesia, and altered sensations, which gradually developed as mixed sensations with allodynia, hyperalgesia, and burning pain, which can be related to “fat worms struggling while burning in fire.” The fever subsided by 3rd May 2021, with a course of Dolo 650 mg; however, there was no relief from the burning pain, heaviness, and psychological distress.

On 3rd May 2021, I was referred to a neurovascular surgeon, and I was diagnosed with left deltoid hematoma and suspicion of CRPS due to wrong injection technique. The doctor had advised intermittent hand rest, ice packs, a course of tramadol for moderate to severe pain (VAS pain score was 7-8/10), short movement of the hand to prevent frozen shoulder, and to avoid lifting heavy objects. Being a practicing dentist, I somehow managed all the patients, who had come for extractions and scaling, single-handed in my clinic. Shortly thereafter, I developed severe pain along the left epicondyle region, involving the left elbow, wrist, middle finger, and ring finger, and was referred to an orthopedic surgeon. After a thorough examination, I was diagnosed with lateral epicondylitis or tennis elbow by mid-June 2021 and was managed with therapeutic ultrasound, ice, and drugs. However, even after courses of neuropathic pain medications and steroid shots, my burning pain aggravated, and it can be explained/related as “sprinkling semi-grinded coarse, spicy red chili powder on boiling concentrated tamarind water” and can also be related to the background theme music and sound design composed for the movement of the Bugs in the Hollywood movie “The Mummy.” After a thorough examination by a senior most neurologist from a renowned hospital, I was diagnosed with CRPS by the end of June of 2021.

Along with physical discomfort, there were also multiple, frequent episodes of psychosocial, spiritual, and emotional components, which included embarrassment and shame on social interactions, guilt and self-blame on taking the vaccine at my workplace, compromised sexual life due to depression and agitations, anger on healthcare professionals, self-pity, lack of self-confidence, and seeking compassionate care [3].

The pain management consultant recommended physical therapy, lidocaine patches, a transcutaneous electrical nerve stimulation (TENS) unit, biofeedback, self-hypnosis, stress reduction, bier blocks, trigger point injections, spinal injections, and spinal cord stimulator as a last resort. Treatment was initiated with topical capsaicin, amitriptyline (25 mg per day), prednisolone (15 mg per day), pregabalin (150 mg per day), acetaminophen (1000 g every eight hours), and physical therapy. Due to continuous chronic total pain, the side effects of steroids, and inclination toward alternative medicine, I had consulted an efficient Ayurveda doctor to combat CRPS by June 2021. I was advised to apply certain medicated oil to massage the affected area along with internal medicines that included “Kashayams,” “Leghyams,” and “Arishtams” to detox and correct the Tri-Dosha Vatta, Pitta, and Kapha. By the end of June, my edema reduced gradually, burning pain subsided, and my quality of life improved without depending on others. I was able to take bath on my own and comb my hair with my fingers for several weeks, and I was able to lift vessels such as cooker and plates with assistance and caution, was able to turn toward my left side while sleeping, was able to drive a manual car by operating the gears, was able to type in mobile, and gradually gained strength to hold a pen while writing prescriptions for my clinic patients. I was also able to raise my hand to switch on the fan (by extra assistance by holding my left upper arm by my right hand) and was able to open my bottle independently. Along with medications, I also improved my willpower and spiritual behaviors by having faith in God and in my future, mindful practice to control and calm down my pain and sensations. Although I have not fully recovered to date, I am able to manage my ADL independently as the CRPS episode in my life strengthened me mentally.

## Discussion

CRPS is purely a clinical diagnosis marked with puzzling pain, disability, and psychosocial and emotional components occurring due to trauma [1,2]. CRPS type 1 is more common and does not involve nerve damage, while CRPS type 2 strictly follows a direct nerve injury. The Budapest Criteria for the diagnosis of CRPS have established four distinct categories (sensory, vasomotor, sudomotor/edema, motor dysfunction and/or trophic changes); however, to date, there is no single “gold standard” diagnostic test [1,2]. A successful outcome relies on early diagnosis and treatment; however, few patients might have a spontaneous resolution. Siegel et al. reported CRPS in animal studies due to needle stick injury causing axonal Wallerian degeneration and decreased epidermal neuritis [4]. Woolf explained that peripheral and central sensitization can lead to continuous chronic pain [5]. Genc et al. reported a case of CRPS post rubella vaccination [6]. Kwun et al. reported a case of CRPS post vaccination of influenza A (H1N1) in a 17-year-old female and suggested that the injection had caused the trauma [7]. Jastaniah et al. reported CRPS in six children of fourth grade post hepatitis vaccination and suggested that the reaction might have resulted from injection trauma [8]. Several other studies explained the possibility of stress and psychological factors in CRPS [9]. I have a stressful work life due to the disparity of income issues at my workplace, and at times, I find it difficult to manage my family life. Therefore, mental stress exists in my situation; however, it cannot be directly attributed to my CRPS. The International Association for the Study of Pain (IASP) presented the best-known criteria for CRPS (Table 1) [6].

**Table 1:** International Association for the Study of Pain Diagnostic Criteria for Chronic Regional Pain Syndrome Type 1 Criteria 2–4 must be satisfied.

Diagnostic Criteria for Chronic Regional Pain Syndrome Type 1	
1	Presence of an initiating noxious event or a cause of immobilization
2	Continuing pain, allodynia, or hyperalgesia with which pain is disproportionate to any inciting event
3	Evidence at some time of edema, changes in skin blood flow, or abnormal sudomotor activity in the region of the pain
4	Diagnosis is excluded by the existence of conditions that would otherwise account for the degree of pain and dysfunction

Hypersensitivity reaction should be included as a differential diagnosis; however, I do not have a history of the same. Studies report that physician education and staff nurse training regarding injection techniques and anatomy can mitigate the incidence of CRPS [10].

In my case, improper injection technique by the staff while administering the COVID-19 shot has initiated the incidence of CRPS with its classic signs and symptoms and not the COVID-19 vaccine itself. However, my CRPS diagnosis does not have an explainable correlation with tennis elbow, as it was an incidental finding due to overload and repetitive motion of wrist and arms due to my profession as a practicing dentist. Eventually, both conditions occurred at the same period, which aggravated my total suffering.

I choose alternative therapy based on trustworthy referrals from my known circles, and it was purely my decision (autonomy). I believe that it is my right to choose or refuse the treatment plan taken on me. However, long-term follow-ups and maintenance are required to assess my overall quality of life. In my case, Ayurveda treatment provided excellent symptom relief and an overall feeling of goodness; however, further extensive research on alternative treatments is required to document substantial evidence of symptom relief for neuropathic pain.

## Conclusions

The wide range of management measures of CRPS includes strong opioids, anticonvulsants, tricyclic antidepressants, free radical scavengers, IV ketamine, baclofen, percutaneous sympathetic blockades, spinal cord stimulation, physiotherapy, occupational therapy, and vitamin C, to name a few. Although the treatment chart involves a multidisciplinary team, my dignity, autonomy, and total suffering should also be addressed and considered as my condition involves complex psychosocial and emotional issues due to its chronic nature, hampering my activities of daily living.

To my knowledge, this is the first study to report CRPS post COVID-19 vaccination shot. Long-term lifestyle modifications and follow-ups are needed to combat chronic suffering to improve the overall quality of life.
